# Population thinking and natural selection in dual-inheritance theory

**DOI:** 10.1007/s10539-012-9307-5

**Published:** 2012-01-26

**Authors:** Wybo Houkes

**Affiliations:** Philosophy & Ethics, School of Innovation Sciences, Eindhoven University of Technology, Eindhoven, The Netherlands

**Keywords:** Cultural evolution, Darwinism, Dual-inheritance theory, Natural selection, Population thinking

## Abstract

A deflationary perspective on theories of cultural evolution, in particular dual-inheritance theory, has recently been proposed by Lewens. On this ‘pop-culture’ analysis, dual-inheritance theorists apply population thinking to cultural phenomena, without claiming that cultural items evolve by natural selection. This paper argues against this pop-culture analysis of dual-inheritance theory. First, it focuses on recent dual-inheritance models of specific patterns of cultural change. These models exemplify population thinking without a commitment to natural selection of cultural items. There are grounds, however, for doubting the added explanatory value of the models in their disciplinary context—and thus grounds for engaging in other potentially explanatory projects based on dual-inheritance theory. One such project is suggested by advocates of the theory. Some of the motivational narratives that they offer can be interpreted as setting up an adaptationist project with regard to cumulative change in cultural items. We develop this interpretation here. On it, dual-inheritance theory features two interrelated selection processes, one on the level of genetically inherited learning mechanisms, another on the level of the cultural items transmitted through these mechanisms. This interpretation identifies a need for further modelling efforts, but also offers scope for enhancing the explanatory power of dual-inheritance theory.

Efforts to extend Darwinism beyond its natural home are almost as old as the *Origin of Species*. Still, announcements of a “Second Darwinian Revolution” (Cziko 1995) seem premature: evolutionary theories of culture and other human-made items remain highly controversial. Several arguments are on offer why basic concepts of evolutionary theory, such as ‘variation’ and ‘inheritance’, can be generalized so that they apply to virtually any complex system (e.g., Dennett 1995). Yet it is widely acknowledged that these concepts should be supplemented with domain-specific details to construct even how-possible explanations (Hodgson and Knudsen 2006)—and it remains unclear how to fill in these details consistently. Conversely, some have argued that there are principled differences between natural and artificial items, such as the high frequency of horizontal transmission in cultural change (e.g., Tëmkin and Eldredge 2007). Yet such arguments may be countered by pointing out similar complications in the evolution of biological items (e.g., Collard et al. 2006).^1^

An interesting perspective on—and perhaps resolution of—these persistent controversies has recently been proposed by Lewens (2009, 2010). Where most of the controversies focus on commitments that theories of cultural evolution need to express in order to count as ‘properly’ or ‘genuinely’ evolutionary or selectionist, Lewens focuses on the commitments that these theories need in order to be of disciplinary interest. Building on Mayr’s (1976) discussion of Darwin’s great contributions to biology,^2^ Lewens argues that the best current efforts to extend Darwinism mainly apply population thinking to cultural phenomena, without necessarily claiming that cultural items evolve by natural selection, or that they are subject to Darwin’s hypothesis of common ancestry. Lewens defends this deflationary view in particular for dual-inheritance theory (henceforth: DIT), as pioneered by Boyd and Richerson (1985):

[Boyd and Richerson]’s insights come from an approach which asks what the population-level consequences might be when we model a group of interacting individuals with characteristic psychological dispositions. They see themselves as inheritors of a Darwinian tradition here, and it is true that Darwin’s account of the modification of species rests on the aggregrated effects of interactions of individual members of those species. Natural selection, although present in some of Boyd and Richerson’s models, is not their explanatory centrepiece; population thinking is. (Lewens 2010: 833)

Let us call this interpretation of theories of cultural evolution, which takes population thinking, not natural selection or common ancestry, to be central to theories of cultural evolution, the ‘pop-culture’ analysis.

If the pop-culture analysis would be correct, it would show that the burden of proof resting on advocates of cultural evolution is significantly lower than one might assume. In particular, attempts to ‘neo-Darwinize’ culture by introducing gene-like memes as units of selection as well as the many criticisms of such attempts would be equally irrelevant to the success of theories of cultural evolution. Moreover, as Lewens points out, advocates of DIT apparently subscribe to the pop-culture analysis. Richerson and Boyd, for instance, state in the introduction to *Not by Genes Alone*, their general-audience presentation of DIT, that “Population thinking is the core of the theory of culture we defend in this book” (2005: 5).

This paper argues against a pop-culture analysis of DIT. After some terminological clarifications and a presentation of the basic structure of DIT, a pragmatic assessment of current work in DIT is given. Some current models that apply insights from DIT to specific patterns of cultural change indeed exemplify population thinking without a commitment to natural selection of cultural items. Yet there are grounds for doubting the added explanatory value of these models in their disciplinary context—and thus grounds for engaging in other potentially explanatory projects based on DIT. One such project is suggested by advocates of DIT in several motivational narratives for their theory. These narratives can be interpreted as setting up an adaptationist project with regard to cumulative changes in cultural items. As a result, DIT may be reconstructed as featuring two intricately related selection processes, one on the level of basic learning mechanisms, another on the level of the cultural items transmitted through these mechanisms. Finally, we consider and refute three varieties of the objection that natural selection is not the kind of process that could operate on cultural items.

## Population thinking and natural selection

To prepare the grounds for our arguments against the pop-culture analysis, it needs to be made clear how and to what extent population thinking can be differentiated from the principle of natural selection.

According to Mayr, who introduced the term, “population thinking” is Darwin’s third and overlooked contribution to evolutionary biology. Although many agree with Mayr on this, the proper specification and precise implications of population thinking are still debated in the philosophy of biology (see Ariew 2008 for an overview). Here, a relatively non-demanding reading is adopted: population thinking minimally involves seeking explanations at the level of populations of varying individuals, rather than that of homogeneous kinds; and acknowledging that the variation between individuals is “causally important” (Godfrey-Smith 2009: 11). Lewens (2009: 249) offers a narrower characterisation of the type of population thinking that he sees exemplified in the work of Boyd, Richerson and others: “the kind of thinking one engages in when one explains the behavior of a unit composed of varied parts in terms of the properties of those parts and their interactions.” This “aggregative thinking”, as Lewens calls it, does not appeal to population-level causal processes, but explains population-level results purely through individual-level processes. On either reading, population thinking can—as Mayr also pointed out—be contrasted with “typological thinking”, a mode of thought which seeks explanations at the level of types or kinds of individuals, and regards variation within such types as idiosyncrasies that are irrelevant for explanations.

Darwin’s Principle of Natural Selection can be characterised as the hypothesis that, under some conditions, a population-level process—“natural selection”—operates in a collection of items that may change trait distributions over generations of items. Three related conditions for natural selection to occur are often given, following Lewontin (1970): that there is variation in the considered trait among items in the population (‘variation’), that this variation influences the reproduction rate of items (‘selection’), and that there is resemblance of traits over generations (‘retention’).^3^ On any specification of the conditions, and arguably also under every interpretation of the process, evolution by natural selection is only one possible process that operates in populations of items, and that is affected by variation among the items. The characterization of natural selection just given is meant to be compatible with natural selection playing explanatory roles besides that in explaining trait distributions (see Stegmann 2010 for an overview); with the non-necessity and non-sufficiency of natural selection for the occurrence of *actual* changes in trait distributions; with both the Negative View of selection and views that accord it a more constructive role; and with both ‘Newtonian’ views of natural selection as a causal force operating on populations (Sober 1984; Millstein 2006) and ‘statistical’ views (e.g., Walsh et al. 2002).

On the—admittedly broad—characterizations just given, the principle of natural selection is one explanatory hypothesis among many that are compatible with population thinking. Thus, the pop-culture analysis is neither ruled out in advance nor necessarily true.

Take, for example, the sigmoid curve that is characteristic of technology diffusion (e.g., Rogers 2003, Ch.7): a slow initial diffusion is followed by a sharp increase in market penetration, and ends in an ever slower approach to maximum market share. One popular model of this curve (e.g., Bass 1969) distinguishes two groups, “innovators” and “imitators”, in a population of consumers. At first, only innovators adopt the new technology. Then, more and more imitators follow suit, until the technology has diffused in the entire population. This model appeals to variations between individuals in a population, and therefore exemplifies population thinking. Yet it involves no differential fitness, or even generations of items, and therefore does not appeal to natural selection. Thus, models and theories can exemplify population thinking without commitment to the principle of natural selection.

Neither population thinking nor giving natural-selection explanations commits one to claiming that items in the population are the result of genetic replication. This replication may be regarded as one inheritance mechanism among many that is compatible with the conditions for natural selection to occur. Therefore, one might, following Godfrey-Smith (2009: 148), distinguish between populational, Darwinian and neo-Darwinian explanations of changes in trait distributions. The pop-culture analysis reconstructs theories of cultural evolution as populational, but not Darwinian, let alone neo-Darwinian. As we shall see, rejection of neo-Darwinian explanations of cultural phenomena (in particular, memetics) provides some of the context for self-presentations and interpretations of the role of natural selection in dual-inheritance theory. This context is apt to mislead, insofar as it suggests a rejection of Darwinian explanations along with neo-Darwinian explanations.

## Dual-inheritance theory: the basics

Dual-inheritance theory (DIT) seeks fruitful explanations of cultural phenomena by positing two related inheritance mechanisms: the genetic-replication mechanism familiar from biology, and a mechanism that transmits cultural traits between individuals who may or may not be related genetically. Culture is defined broadly, as any type of information that is acquired from other members of one’s species and that might affect behaviour (e.g., Boyd and Richerson 1985: 33; Richerson and Boyd 2005: 5). Culture therefore includes laws, gossip, technology, skills, scientific theories, rules of etiquette and feeding habits—henceforth called ‘cultural items’ or ‘cultural traits’. No attempt is made to argue that transmission of cultural items is like genetic replication. Indeed, modelling efforts show that transmission may involve blending of traits, which are not discrete packages of information; yet that they can sometimes be represented as if they were (Henrich and Boyd 2002).

The general approach taken in Boyd and Richerson (1985), the seminal work of DIT, is to model changes in cultural traits over generations analogously to phenotypic changes of biological items. This is taken as warrant to apply to cultural phenomena many of the modelling techniques developed by population biologists. In this vein, DI theorists model the spread and effects of ethnic markers; the spread and effects of social learning mechanisms and various ‘content and context biases’, i.e., psychological mechanisms that aid and constrain learning by making human beings prone to learn specific things (e.g., simple melodies rather than superstring theory) from specific people (e.g., media personalities rather than homeless doomsayers).^4^

The importance of these social learning mechanisms in DIT can hardly be overestimated. As stated above, the definition of culture appeals to social learning, meaning that some basic mechanisms of social learning cannot be a part of culture and subject to its distinctive transmission mechanisms: we are not encultured to be learners of a specific type, but we can be encultured because we are learners of a specific type. In effect, the learning mechanisms make explicit what cultural transmission amounts to, and which constraints it has. Basic mechanisms such as conformity and prestige bias^5^ enable the emergence of a great diversity of cultures, but that they themselves operate more or less invariably in *all* human cultures, from that of Tasmanians to that of the Dinka and American college teachers. This, according to DI theorists, partly answers what they memorably call the “Why not baboons?” question: why, of all species on this planet, do only human beings have rich cultural systems? That these learning mechanisms are a generic result of human evolution is a decidedly non-trivial claim. It is supported by models that represent how mechanisms such as prestige, success and conformity bias could have evolved by natural selection, despite such disadvantages as learning costs, increased brain size/caloric intake, and substantial error rates.

This executive summary fits some programmatic statements, much of the actual work and virtually all modelling efforts in DIT. Moreover, it fits the pop-culture analysis to a T, including the role of natural selection in some models (i.e., those of the evolution of social learning mechanisms) as mentioned by Lewens. Yet, as presented above, DIT is a theory of the evolutionary origin of social or cultural learning in (all) human groups. It is not a theory of culture as defined above, since the basic cultural learning mechanisms or ‘forces’ (such as content and context bias) are not themselves transmitted by cultural learning.^6^

At least two strategies are available to apply DIT to more specific patterns of cultural change, such as the diffusion of agricultural practices or the diversification of religious beliefs and tool traditions. One, relatively straightforward strategy is to model directly how characteristic patterns of cultural change could result from basic learning mechanisms such as prestige and conformity bias. Another, more circuitous strategy is to consider the interaction between specific patterns of cultural change and the evolution of basic learning mechanisms.^7^ In the next section, we argue that the attractions of the former route should not be overrated. Then, we present a case for the second route, based on motivational narratives presented by DI theorists, and argue that natural selection of cultural items might play an explanatory role in it.

## Explaining cultural change by social learning: a pragmatic evaluation

At the end of the previous section, two strategies were sketched for applying DIT to specific patterns of cultural change. In this section, we consider the first strategy, which is to model how such patterns could arise from the basic social learning mechanisms identified in DIT. Now the potential area of application of this strategy is enormous, and evaluation of the explanatory merits of individual models may be largely an empirical matter. Still, it is possible to indicate, in general, how DI models of specific cultural phenomena have to prove their worth—namely by correcting existing explanations and/or providing fresh insights in a disciplinary context. As it is put in one leading critique of models of cultural evolution:

… the question about the usefulness of these models of cultural evolution to the day-to-day research of social scientists comes to this: Are social scientists good at intuitive population thinking? If they are, then their explanations will not be undermined by precise models of cultural evolution. (Sober 1991, n.18)

Several candidate theories of cultural evolution fail to pass this pragmatic “disciplinary competitiveness” test. To give one example: as an explanatory theory, Basalla (1988) evolutionary account of technological change only amounts to a rejection of “Great Man” views—which no contemporary historian of technology takes to be viable anyway (Lewens 2004, Ch.7; 2009). More pertinently, Sober himself discusses DIT in this context,^8^ and Lewens (2009) defends the usefulness of the theory and other examples of population thinking on cultural phenomena.

In this context, Lewens indirectly^9^ refers to two models for patterns of technological change, constructed by DI theorist Joseph Henrich, as well as to some non-DIT population-dynamical models of cultural change (e.g., Nelson and Winter 1982). Henrich (2001) presents a model for the characteristic sigmoid curve of technology diffusion, showing that it could arise from the operation of cultural learning rules, in particular prestige and conformity bias, and is unlikely to arise without the operation of these biases. Henrich (2004) models the effects of cultural transmission on technological complexity. It focuses on intergenerational learning of skills and analyses how large the population needs to be to compensate for the constant erosion of skills through imperfect learning. More specifically, it explains how the Tasmanians could have lost much of their technological toolkit after isolation from the cultures of mainland Australia. Both models exemplify population thinking, in focusing on relevant differences in or features (sizes) of human populations, and apply insights from DIT in focusing on the effects of basic social-learning mechanisms. Neither model features the operation of natural selection on populations of cultural items. Therefore, these DI models of cultural change fit the pop-culture analysis just as well as the basics of DIT summarized in the previous section.

Both models have aroused substantial interest, most notably in anthropology and archaeology.^10^ Therefore, one might take them to pass the “disciplinary competitiveness” test effortlessly. However, a more careful assessment of each model shows that both illustrate not only some potential rewards of directly modelling cultural change with DIT, but also some risks of this first strategy.

Let us start with Henrich’s (2001) model of technology diffusion. A brief look at some relevant literature shows that the relevance of this model within its disciplinary context (anthropology) resides in large part in transferring insights that have long been available in other disciplines. For in innovation studies and technology forecasting, there is a variety of models for technology diffusion, all of which reproduce the characteristic sigmoid curve. Three model families—Fisher and Pry (1971) substitution models, the Bass (1969) innovator-imitator models briefly discussed above, and various obsolescence models—are both scientifically and commercially well-established; so much so in fact that technology-forecasting research after the 1980s has largely focused on applications, refinements and hybridizations of these models. Against this background, Henrich’s model either competes with the existing models, or is a social-learning reformulation of the Bass model, explaining innovators’ and imitators’ differential dispositions in terms of their differential success biases. In his discussion of the literature, Henrich mainly refers to Bass’ (1969) remark that existing models lack a firm theoretical foundation. In three decades of diffusion research, however, this remark has lost some of its ground. If Henrich’s model seeks to provide the Bass model with a “foundation in human psychology” (2001: 1006), it not only leaves the Bass model intact, but also just puts it on an equal footing with the Fisher-Pry model, which has been grounded in models of competition and substitution mechanisms (e.g., Porter et al. 1991; Morris and Pratt 2003). In case Henrich’s model would be more than a reformulation of the Bass model, i.e., if the model shows different behaviour from that of its rivals (now including the Bass model), it remains to be seen whether these could be discerned in empirical datasets: it is generally acknowledged in the technology-diffusion literature that differences between the Bass, Fisher-Pry and obsolescence models—which include early adoption rates and (a)symmetry around the inflection point—are too subtle and empirical datasets too limited to allow significant testing.^11^

Evaluating the explanatory value of Henrich’s (2004) model in its disciplinary context is slightly more involved. Unlike the technology-diffusion model, it focuses on a particular episode of cultural change, namely loss of tool complexity among Tasmanians. It offers a clear alternative to existing how-possible explanations, which appeal to, for instance, cultural drift or adaptive decision making.^12^ A selection of recent related work directly speaks in favour of the (multi-) disciplinary relevance of Henrich’s “Tasmanian tool-loss” model. However, it also shows how complicated evaluation of the explanatory value of the model is, and how it might fail to deliver on some or all promises.

Firstly, on the *explanans* side, it has not remained uncontested. Some have pointed to anthropological counterevidence, in which small populations maintain highly complex technologies (Read 2006; see Henrich 2006 for a response). Others have pointed out that the population-size effect is not nearly as pronounced if some of the model’s assumptions are changed for more realistic ones (Bentley and O’Brien 2011). Conversely, on the *explanandum* side, still others have extended the explanatory scope of the model, by using it to explain sudden *increases* in cultural complexity, in particular in the late Pleistocene (Powell et al. 2009), or to explain scientific progress in the face of structural cognitive biases (De Cruz and De Smedt 2010). Here, however, the assumption of perfect mentor selection, made by Henrich for tractability purposes, might undermine the explanatory value of the model.^13^ Besides in terms of robustness and empirical adequacy, the model can also be evaluated in terms of compatibility with more general, non-DIT models. One such model (Enquist et al. 2011) explains characteristic patterns of technological change, such as diversification and stepwise modification, in terms of dependencies between technologies, and therefore without assuming any specific learning mechanisms.

Given the diversity of applications, amendments and attacks, a full evaluation of the disciplinary relevance of the Tasmanian tool-loss model shall not be attempted here. It is clear, however, that this evaluation is partly a matter of assessing whether aggregative or population thinking is a familiar mode of explanation within the field. Some alternatives, such as adaptive decision-making models, do not employ population thinking, whereas others, such as the technological-dependencies model of Enquist et al. (2011), do. The latter, moreover, might include Henrich’s model as a special, psychologically grounded case—roughly analogous to the relation between the Bass (1969) and Henrich (2001) models of technology diffusion. Finally, every assessment is complicated by the lack of agreement over what can and should be explained under the heading of ‘cumulative’ cultural change—a topic to which we shall return in the next section.

Summing up, in this section, some reasons were given to be cautious about the explanatory value of both of Henrich’s models. These do not amount to arguments for abandoning the strategy of constructing DI models for patterns of cultural change, let alone for rejecting any theory of cultural evolution. Rather, they show that DI theorists might want to ‘spread their risks’, and invest some of their modelling efforts in the second, indirect explanatory strategy. The next section presents one way to implement this strategy, which is not only suggested in some narratives used to motivate DI theory, but that also contributes to specifying—in terms of natural selection—the phenomenon of cumulative cultural change .

## Cumulative cultural change and explanatory adaptationism

Earlier, two strategies were sketched for applying DIT to patterns of cultural change: one that directly models them as resulting from basic social-learning mechanisms, and another that focuses on the interrelations between the evolution of learning mechanisms and patterns of cultural change. Then, some grounds were given for being cautious about the explanatory merits of models that implement the first strategy. These do, of course, not entail that advocates of DIT should invest *exclusively* in the second strategy. In this section, we show one way of implementing the second strategy that is complementary to the first strategy.

This implementation starts with some compelling motivational narratives given by DI theorists. There, attention is drawn to a vital characteristic of human cultures, namely that the great diversity of cultural items, such as hunting practices, technologies and dietary restrictions, have enabled human beings to thrive in virtually every corner of the globe, including inhospitable areas like arctic coasts and inland deserts. Moreover, the motivation continues, these cultural adaptations cannot be the result of individual inventiveness. As Henrich and McElreath (2003) describe vividly in their presentation of the basic structure of DIT, neither advanced technology nor prolonged exposure to the local environment helped Robert Burke’s 1860 expedition to cross the Australian desert. Richerson and Boyd (2005: 46–48) use the similar example of crossing El Camino del Diablo between California and Mexico, the unwelcoming home of the Papago Indians. From these examples, DI theorists conclude that human beings can thrive in various environments, not because they are exceedingly clever adapters individually, but because they have acquired suitable cultural repertoires over many (human) generations.

These motivational narratives set up a particular explanatory project, which shall be called the ‘cumulative-adaptation’ project here. Its explanandum is the acquisition, by groups of humans, of local cultural adaptations such as hunting practices and tool traditions. Its explanans is characterized both positively and negatively: it involves the gradual accumulation of an adaptive cultural repertoire over many generations and it *does not* involve individual inventions of optimal cultural items.

This project is not marginal to DIT. It specifies the “Why not baboons?” question alluded to earlier, by clarifying in which respects and to what extent human beings are unlike baboons. Earlier, part of the answer was given: human beings are capable of cultural learning, which is characterised by particular transmission mechanisms (‘forces’ or ‘biases’) and which can arise as the result of (multi-level) natural selection on human beings. By this type of learning, human beings are capable of imitating a clever trick after they have observed a conspecific employing it.

However, the narratives just given go beyond noticing this imitative capacity. They point out that humans are capable of retaining clever tricks *and* gradually modifying them to a level of ingenuity that no individual inventor could ever hope to achieve.^14^ To illustrate how this offers a more elaborate view on how human beings are unlike baboons, consider cycling. We transmit the cultural trait of cycling over the generations by cultural learning—and substantial time and patience on the teacher’s part, and willingness to cope with frustration and occasional pain on the student’s part. Baboons are incapable of this.^15^ This means that, even if—which is conceivable—the trick of riding a bicycle is within the grasp of a socially isolated individual learner, the trick would die with its baboon inventor, but is likely to outlive its human inventor. In addition, human beings are unlike baboons in being able to design bicycles, which have thousands of integrated parts and embody technological knowledge of huge complexity. In combination, these two uniquely human features characterize how we are unlike baboons, and thus specify one of the central questions posed by DI theorists.

The cumulative-adaptation project set up by the motivational narrative not only specifies the “Why not baboons” question. It also strongly resembles what Peter Godfrey-Smith (2001) has called *explanatory adaptationism*, both in its explanandum and in its rejection of a particular candidate explanation. In ‘biological’ explanatory adaptationism, the ‘Big Question’ is to explain the adaptedness of life forms to their environments without appealing to the perfect wisdom of an individual Designer of the life form. The ‘Big Answer’ lies in the cumulative changes wrought by the operation of natural selection on life forms. In DIT, the Big Question is the adaptedness of (groups of) human beings to their local environments, and the Big Answer is supposed to lie in the cultural transmission and gradual accumulation of cognitive capital over many generations, without appealing to the wisdom of an individual designer.

Given the resemblance of this project to explanatory adaptationism, part of the answer to the “Why not baboons?” question might lie in natural-selection explanations on the level of cultural items, as well as on the genetically inherited capacities for cultural learning. The former natural-selection explanations would involve populations of cultural items (say, arrowheads) that are replicated through production, use, maintenance and other human activities. To give a semi-specific example: the shape of arrowheads may change over generations, under the influence of learning mechanisms such as success and prestige bias, and despite the influence of other mechanisms, such as conformity bias. Moreover, small variations are introduced by individual intent or accident. Some of these may be detrimental to the successful use of the item; others may increase its usefulness for present purposes, or may play a role in alternative uses. As a result, generations of arrowheads might arise as adaptations, showing the gradual refinement and diversification that is characteristic of human cultural traditions (Richerson and Boyd 2005: 115).

Not every suite of social learning mechanisms facilitates this pattern of cumulative cultural change. If human learning would, for instance, be dominated by conformity bias, small innovations in design or use would never spread, no matter how advantageous they would be.^16^ Without success and prestige bias, there would be no retention of the cultural repertoire over multiple generations; without imitation errors and (limited) individual inventiveness, variation in cultural traits would quickly disappear. Yet that certain varieties of these learning mechanisms, in combination, allow adaptive technologies to accumulate might be pivotal in explaining how *these* (combinations of) varieties evolved by natural selection: the evolutionary benefits of the former reside in enabling the latter. As Richerson and Boyd (2005:7) put it: “the human cultural system arose as an adaptation because it can evolve fancy adaptations to changing environments”.

In this ‘dual-selectionist’ interpretation, DIT involves two coupled selection processes: one that acts on populations of human beings and explains how they have evolved specific learning mechanisms; another that operates on populations of cultural items and explains how they could be optimized to a great diversity of environments. The first part of this explanation cannot be divorced from the second: human beings evolved their unique suite of social learning mechanisms *because* it enables, under certain conditions and in some cases, the gradual accumulation of cultural capital. Conversely, an explanation of this cultural accumulation cannot be divorced from an explanation of social learning mechanisms.^17^ This interrelation resembles that between more specific examples of gene-culture co-evolution, such as that between the spread of lactose tolerance and of dairying practices.

Boyd and Richerson occasionally hint at these interrelated selection processes, e.g.: “The single most important adaptive feature of culture is that it allows the gradual, cumulative assembly of adaptations over many generations, adaptations that no single individual could invent on their own.” (Boyd and Richerson 2000, p.424). Here, both the learning mechanisms and the products of these mechanisms are referred to as adaptations—and the “generations” referred to could be of either human beings or cultural items.^18^ Elsewhere, an intricate interaction between the evolution of complex cultural traditions and the evolution of ever more sophisticated learning mechanisms is suggested: “As the evolving traditions become too complex to imitate easily, they will begin to drive the evolution of still more-sophisticated imitation.” (Richerson and Boyd 2005: 139).

As said at the end of the previous section, cumulative cultural change is a complicated and diffuse phenomenon. This section makes clear which aspect of this phenomenon could be particularly relevant as an *explanandum* for DIT. Moreover, its emphasis on the role of social learning mechanisms in acquiring cultural traits appears to provide DIT with the conceptual equipment for giving this explanation. Still, making explicit this explanatory project reveals a hiatus in current modelling efforts. To see why, consider again Henrich’s (2004) tool-loss model, discussed in the previous section. Contrary to the motivational narratives summarized at the start of this section, Henrich’s model assumes unlimited individual inventiveness: every individual draws a skill-level value from the interval [0,∞), albeit with a probability distribution that depends partly on the skill-level value of the individual’s mentor and that is skewed towards a decrease in skill level. Therefore, even if Henrich’s model is taken as explaining cumulative cultural change (rather than the loss of cultural complexity), it does not feature the kind of cumulative change that is presented in the motivational narratives.^19^

This interpretation therefore identifies a considerable burden of proof resting on future DI work. Perhaps this burden cannot be shouldered. In culture, the Big Answer of explanatory adaptationism might be false, just as the Big Question of the cumulative-adaptation project might be mistaken. However, a philosophical analysis of a theory that advises its advocates against endorsing a possibly false hypothesis is clearly too risk-aversive. Engaging in the cultural-adaptation project seems a risk worth taking: it might increase DIT’s explanatory value, and would give it a pivotal role in an evolutionary synthesis of the social sciences (Mesoudi et al. 2006; Mesoudi 2011b: Ch.10).

## Three objections to natural selection of cultural items

In closing, we consider a seemingly fundamental objection against the explanatory project presented above, namely that natural selection is not the kind of process that could operate on cultural items. In this section, three varieties of this objection are discussed and found inconclusive.

In the previous section, we identified an explanatory project of DIT as involving two processes of natural selection, one on the level of genetically inherited capacities for social learning, the other on the level of culturally transmitted items. Taking this project as central to DIT is not just at odds with the pop-culture analysis, but also appears to contradict some self-presentations of DI theorists. Take, for instance, the passage quoted approvingly by Lewens: “population thinking, *not natural selection*, is the key to conceptualizing culture in terms of material causes” (Boyd and Richerson 2000, p.421; emphasis added).

The context of this passage, and others like it, is the rejection of *neo*-Darwinian (i.e., memeticist) theories of cultural evolution. Boyd and Richerson argue against such theories by pointing out several dissimilarites between cultural transmission and genetic replication. However, these arguments only entail that natural selection cannot operate in cultural transmission if genetic replication is taken as a necessary condition for natural selection. Philosophers of biology have, however, offered various reasons for believing that such a gene- or replicator-centered view of natural selection is too strict even in biology (see, e.g., Godfrey-Smith 2009, Chs. 4–7). Thus, rejecting natural-selection explanations of culture along with memetics would commit DI theorists to a contentious view of biological evolution—a commitment that those interested in developing a theory of cultural evolution might best avoid.

Another version of the objection seems to avoid both gene-centrism and a commitment to natural selection of cultural items. Various advocates of theories of cultural evolution have, following Cavalli-Sforza and Feldman (1981), distinguished *natural selection*, which operates on biological items, from *cultural selection*. Here, they characterize the latter as any process that results in differential rates of transmission of cultural traits (e.g., Shennan 2002: 35; Mesoudi 2011b: 64–65). This emphasizes a central similarity with natural selection, while leaving room for the many differences between biological and cultural change. From this perspective, an interpretation of DIT that appeals to natural selection of both learning mechanisms and cultural items might seem to ignore these differences between cultural and natural selection by over-generalizing the latter process.

Although there is some ground to this objection, it misses a central element of the ‘dual-selectionist’ interpretation presented in the previous section. On it, cultural change involves many processes that result in differential transmission of cultural traits. Some of these involve natural selection of the bearers of cultural traits, i.e., human beings; celibacy, for instance, negatively affects an individual human being’s chances of reproduction. Other processes may involve natural selection of cultural items, resulting in cultural adaptations. Still other processes may not involve natural selection in any sense, e.g., technology diffusion that is driven by obsolescence or marketing mechanisms (see Sect. 1). All of these processes may be called ‘cultural selection’, but the crucial point of the dual-selectionist interpretation appeals to the second type, which resembles natural selection both in resulting in differential transmission rates *and* in adaptations. Still, some might insist on making a sharp terminological distinction between these processes and ‘natural selection’.^20^ For them, the dual-selectionist interpretation could be rephrased as featuring an interrelation between a process of *natural* selection operating on genetically inherited and a *specific kind of cultural selection* operating on cultural items and resulting in cultural adaptations.

A third and final version of the objection is to maintain that, given its nature, natural selection cannot be explained or modelled as the result from the operation of learning mechanisms or any other ‘force’ that affects individual items within a population. On the statistical interpretation of natural selection favoured by Lewens (2004; Walsh et al. 2002), aggregative thinking seeks explanations in terms of individual-level selective forces, not in terms of the purely population-level ‘force’ of selection. Thus, natural-selection explanations (on Lewens’ construal) are not a subtype of aggregative thinking, although they are a subtype of population thinking in the broader sense. Therefore, the pop-culture analysis saves DIT from rejection by Lewens—or saves Lewens from having to reject DIT as resting on a mistaken view of selection.

In response to this objection, one could distinguish between reconstructions that favour one particular notion of natural selection, coupled to a particular explanatory practice, and those that are more liberal. This might bring to mind Godfrey-Smith’s (2009) analysis of evolutionary explanations. He distinguishes paradigmatic examples of Darwinian populations that satisfy “core” notions of replication and natural selection from more marginal ones. While arguing that our understanding of some (certainly not all) biological phenomena fits the core notions, he leaves room for scientifically fruitful applications of marginal notions.^21^

The dual-selectionist interpretation offers scope for enhancing the explanatory power of DIT, but it employs a liberal notion of natural selection (or a specific natural-selection-like process of cultural selection) to specify the central explanatory hypothesis. The pop-culture analysis limits DIT to models that explain directly how patterns of cultural change arise from basic social learning mechanisms, and Lewens’ statistical interpretation of natural selection justifies these limitations. Which one to prefer may, for those without strong previous commitments regarding the nature of selection, therefore be a matter of choice between a progressive and a conservative interpretation. On the former, part of DIT’s central explanatory project might be misguided; on the latter, its empirically oriented models may not pass the ‘disciplinary competitiveness’ test. Identifying these risks may be a job for philosophy of science, choosing to avoid either or neither of these risks is definitely that of science.

## Footnotes

1 Such counterarguments may get additional fuel from calls for a more inclusive philosophy of biology (O’Malley and Dupré 2007), since many of the supposed idiosyncrasies of cultural change have counterparts in micro-organisms or, more broadly, non-mammalian organisms.

2 Mayr discerns three contributions: Darwin proposed the Principle of Natural Selection (or descent with modification), gathered evidence for the operation of this principle in the organic world, and replaced ‘typological thinking’ with ‘population thinking’. Many authors have followed Mayr in distinguishing three contributions or Great Ideas, where the evidence-gathering is often replaced with the hypothesis of common ancestry. This hypothesis is, on another argument of Mayr’s (1982), conceptually independent of the Principle of Natural Selection, although their evidential relations are quite tangled (e.g., Sober and Orzack 2003).

3 These conditions are neither necessary nor sufficient, but sufficiently precise for our present purposes. See Godfrey-Smith (2007) for modifications of and amendments to the Lewontin conditions.

4 Boyd and Richerson (2005) contains many of these results.

5 The explanation of various basic mechanisms, including a classification of types, is usefully summarised in Henrich and McElreath (2003). They only mention natural selection operating *on genes* to produce social-cultural learners like ourselves. This exemplifies the narrow concept of natural selection that is also used occasionally by Boyd and Richerson (see the final section).

6 DI theorists sometimes use ‘culture’ to refer to the universal human capacity for social and cultural learning rather than to any local cultural traits or traditions. ‘Basic’ DIT is a theory of culture in this alternative sense.

7 A third route, not considered here, would be to derive psychological constraints on cultural transmission that arise from our genetically inherited mental infrastructure. It is not immediately evident that DIT provides constraints of this kind, nor that it should—given the forceful arguments that philosophers have raised concerning evolutionary psychology (e.g., Buller 2005; Richardson 2007).

8 Sober’s main argument is that DI models of the evolution of cultural learning, mentioned earlier in the main text, address a different topic than standard explanations in the social sciences: they explain how cultural learning could have evolved, not how systems of cultural items change (“how cultures work”, Sober 1991). In his more positive assessment, Lewens refers to those DI models, developed after 1991, that *do* address changes in cultural items.

9 Lewens (2009) refers to Richerson and Boyd (2005), who briefly and informally discuss Henrich’s results.

10 Of the 82 citations of Henrich (2001) listed by Web of Science (accessed December 2011), most are in anthropology (24), archaeology (16), behavioural sciences (14) and biological psychology (14). The 98 citations of Henrich (2004) are also mainly in anthropology (39) and archaeology (32), as well as in biology (22) and geosciences (11).

11 Young (2009) concludes his review of several diffusion models by listing a few principled shortcomings of datasets. Kandler and Steele (2010) analyse the merits of Henrich’s (2001) model, arguing that it typically reduces to the Bass model, and include a case study.

12 Henrich (2004: 205–208) rejects adaptive decision-making explanations as an inferior rival, and accepts cultural-drift explanations as a possible complementary.

13 Henrich’s Tasmanian tool-loss model is based on two assumptions: (1) that skill levels in a population follow a Gumbel distribution; (2) that everyone in a population is capable of selecting as a mentor the individual with the highest skill level of his/her generation. Relaxing this second assumption, which is clearly an idealization, means that many students select sub-optimal mentors and that, consequently, the population is less capable of retaining a high maximum skill level. Bentley and O’Brien (2011) discuss the effects of this relaxation, referring to Henrich’s (2004) original application and that of Powell et al. (2009).

14 Richerson and Boyd write that “[t]he history of technology shows that complex artifacts such as watches are not hopeful monsters created by single inventors. The watchmakers’ skills have been built up piecemeal by the cumulative improvement of technologies at the hands of many innovators” (Richerson and Boyd 2005: 51) and “most of the cultural artifacts that we use, the social institutions that shape our lives, the languages that we speak, and so on are far too complex for even the most gifted innovator to create from scratch.” (*ibid*., 53). In their discussion, they use clocks, compasses, churches, ships, religious systems and languages as examples of cumulative culture *(ibid*., 52-54), immediately before introducing the Why not baboons? question and suggesting that culture provides the answer (*ibid*., 55-57).

15 See Whiten et al. (2009), Tennie et al. (2009) and Vaesen (2011) for overviews of the differences in culture, cognition and tool use between human and primates.

16 Henrich (2001: 1002–1003) shows that diffusion of technological innovations only occurs if the ‘relative weight’ of conformity bias in cultural transmission is small.

17 The evolution of social learning mechanisms may partly proceed through cultural group selection (Boyd and Richerson 2009).In the order of explanation presented in that paper, cumulative cultural change—as a constellation of accumulated technologies and learning mechanisms—comes first, however: “We hypothesize that this new social world, created by rapid cultural adaptation, led to the genetic evolution of new, derived social instincts” (ibid., 3286). Still, the full answer to the “Why not baboons?” question is likely to involve three selection processes. Therefore, it may be even more complicated than that presented in the main text.

18 Similarly, ‘cumulative culture’ may refer to both an accumulation of cultural traits, such as tool traditions, and the suite of social learning mechanisms responsible for this accumulation. This reflects an ambiguity of the term ‘culture’ in DIT, as noted in n.6 above.

19 Mesoudi (2011a) has very recently constructed a model of cumulative culture that includes the basic learning mechanisms of DI. Like the Tasmanian tool-loss model, Mesoudi’s model is primarily aimed at identifying conditions (viz. increasing learning costs) under which cultural accumulation ends; yet unlike Henrich’s model, it puts a strict maximum on any individual’s increase in skill level. Therefore, it goes some way towards modelling cumulative culture as presented in DIT’s motivational narratives. Further scope for modelling this process may be found in the more inclusive (and non-DI) technological-dependencies framework presented by Enquist et al. (2011).

20 One reason for this insistence might be a gene-centred view of natural selection.

21 Godfrey-Smith draws examples from biology, not from disciplines involved in the second Darwinian revolution (economics, archaeology, anthropology, etc.).
